# Patient-Reported Outcomes in Pleural Effusions: A Systematic Review

**DOI:** 10.7759/cureus.52430

**Published:** 2024-01-17

**Authors:** Eleanor K Mishra, Andrew Stanton

**Affiliations:** 1 Respiratory Medicine, Norfolk and Norwich University Hospitals NHS Foundation Trust, Norwich, GBR; 2 Faculty of Medicine and Health Science, University of East Anglia, Norwich, GBR; 3 Medicine, Newcastle Upon Tyne Hospitals NHS Foundation Trust, Newcastle, GBR

**Keywords:** patient-reported outcome measure, pleural effusion, quality of life, prom, breathlessness, malignant pleural effusion

## Abstract

Pleural effusions cause breathlessness, decreased activity levels, and impaired quality of life. Clinical trials of drainage of pleural effusion use patient-reported outcome measures (PROMs) to assess these variables. This systematic review aimed to identify which PROMs have been used in clinical trials in pleural effusions, what variables were assessed, whether they were responsive to pleural interventions, and whether they have been validated in patients with pleural effusions, including a defined minimal clinically important difference (MCID). A systematic review was performed to identify relevant clinical trials from Medline, EMBASE, Emcare, and CINAHL and data were extracted.

From 329 abstracts, 29 clinical trials of pleural effusion drainage that used PROMs as an outcome measure were identified. A total of 16 different PROMs were used. The most used PROMs were unidimensional measurements of breathlessness, particularly the visual analogue scale for dyspnoea (VASD), all of which nearly showed improvements in breathlessness following pleural fluid drainage. Other variables commonly assessed included activity levels and health-related quality of life. Multidimensional PROMs showed inconsistent responsiveness to pleural fluid drainage. Only the VASD was validated in this patient group with a defined MCID.

A range of PROMs are used in clinical trials of pleural fluid drainage. No single PROM measures all the outcomes of interest. Unidimensional measurements of breathlessness are responsive to pleural fluid drainage. Only the VASD is validated with an MCID. There is a need for properly validated, response PROMs which measure the key outcomes of interest in this patient group.

## Introduction and background

Pleural effusions are the accumulation of fluid in the pleural space [1]. They are commonly caused by advanced systemic diseases, such as cancer and heart, liver, and kidney failure. These conditions are associated with disabling symptoms and poor prognosis. The effusion is drained to palliate symptoms and make a diagnosis, with the hope of enabling the patient to be more active and improving the quality of life. Usual management is an initial large-volume drainage (therapeutic thoracocentesis (TT)), followed by definitive management (indwelling pleural catheter (IPC) or pleurodesis). Pleural effusions can also be caused by infection, but these are not the focus of this review because the goal of treatment is cure and important outcomes are death and need for surgery.

For use in clinical trials, a patient-reported outcome measure (PROM) must be validated in this patient group and responsive to pleural interventions [2]. It must ask about the symptoms and variables which are important to patients and clinicians. Observational studies demonstrate that the symptom most commonly reported by patients with pleural effusions which improves with intervention is breathlessness [3]. Other symptoms experienced include cough, chest pain, poor sleep, fatigue, and anorexia. Cough, pain, and sleep quality sometimes improve following drainage. Some symptoms (e.g., fatigue, anorexia) may be due to the underlying systemic disease rather than the pleural effusion and, therefore, will not improve with drainage. Other important outcomes for clinicians are health-related quality of life and activity levels. There are many validated PROMs which assess these symptoms, such as the visual analogue scale (VAS), which is used for breathlessness and pain, the Brief Pain Inventory, Dyspnea-12, and the Borg scale for exertion-induced breathlessness [4-8].

The primary aim of this systematic review was to identify which PROMs have been used previously in published clinical trials of patients with pleural effusions. Secondary aims were to identify what variables were assessed in these PROMs; whether the PROM was responsive to pleural intervention; and, finally, whether they have been validated for use in this patient group, including a defined minimal clinically important difference (MCID, the smallest change in a measurement which patients consider worthwhile for a specific intervention) for patients with pleural effusions. The longer-term goal of this work is to identify whether there is an ideal PROM to assess long-term symptoms in patients with pleural effusions secondary to cancer or organ failure. Given the focus on long-term symptoms, we excluded studies which only assessed pain around the time of a pleural procedure.

## Review

Methodology

This systematic review adheres to Preferred Reporting Items for Systematic Reviews and Meta-Analyses (PRISMA) guidelines. A protocol for the study was written before the literature search (available from the authors). This was not registered due to the absence of an appropriate online repository. Medline, EMBASE, Emcare, and CINAHL were searched on January 27, 2023, for clinical trials using the search terms ‘pleural effusion’, ‘drain’, ‘catheter’, and/or ‘pleurodesis’. Only studies published in English were included. Duplicate articles, conference abstracts, and papers which used PROMs only to assess the pain of the procedure or pleurodesis were excluded.

Identified abstracts were screened for relevance by AS and EM independently to identify clinical trials and prospective observational studies which used a PROM as an outcome measure. Differences were resolved by reviewing the complete paper. Potentially relevant papers underwent full-text review by both authors. Data from relevant papers on trial type, PROMs used, and whether the PROM was responsive to the intervention was extracted from all papers by either AS or EM independently and reviewed by both authors. Specifically, data on whether the PROM was responsive to any intervention compared to baseline was extracted, not whether one intervention showed evidence of benefit over another. No study investigators were contacted to obtain additional information. Given that this study aimed to identify PROMs used in clinical trials and prospective observational studies, no assessment of bias was done in the included studies. A subsequent literature search was conducted for all identified PROMs to assess whether they had been specifically validated in patients with pleural effusions and whether there was a defined MCID specifically defined for patients with pleural effusions.

Results

**Figure 1:**
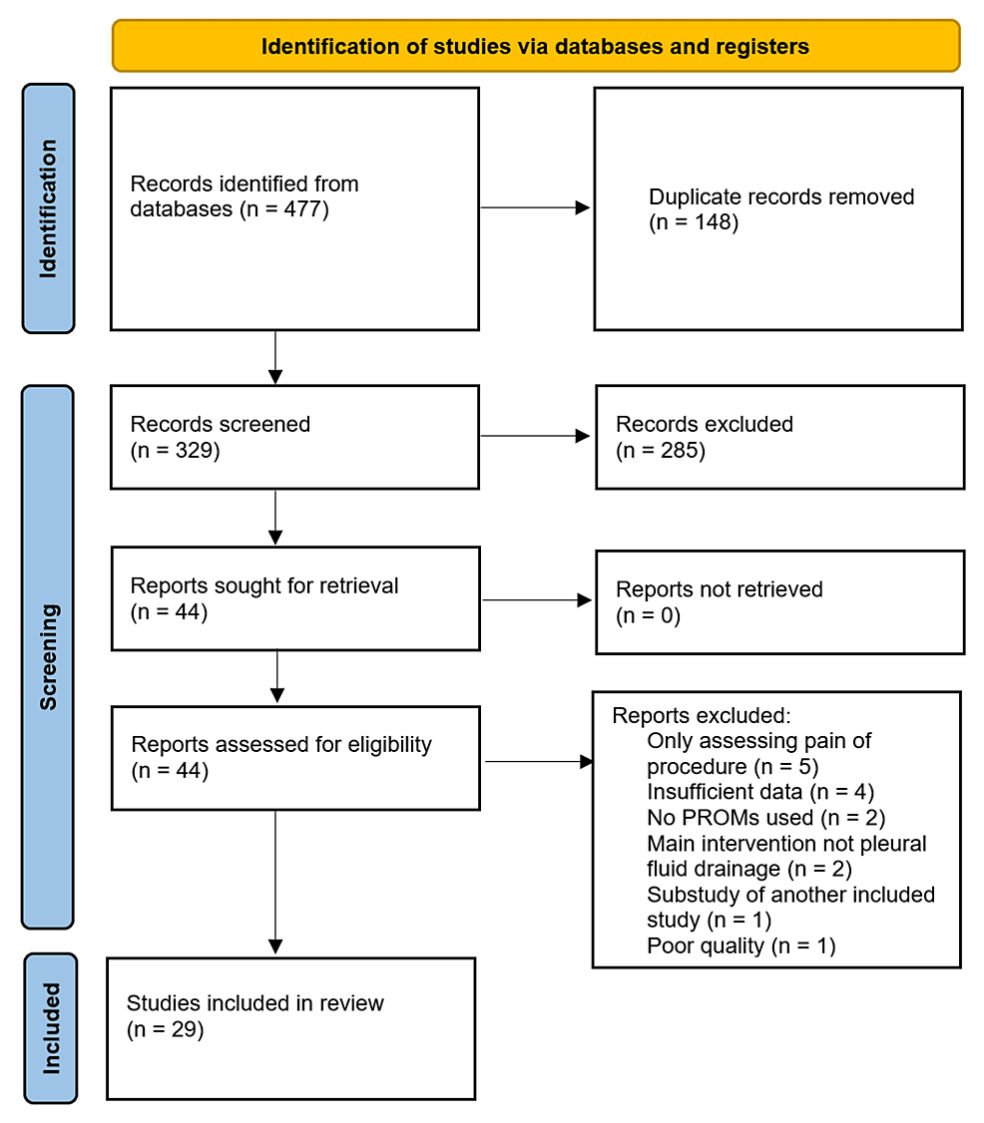
Preferred Reporting Items for Systematic Reviews and Meta-Analyses (PRISMA) flowchart of the studies reviewed.

After the removal of duplicates, 329 abstracts were identified in the initial search. Of these, 44 papers were chosen for full-text review, and 29 papers were included in the analysis (Figure 1).

Of the 29 relevant studies identified, 19 were randomised controlled trials, one was a non-randomised controlled trial, and nine were prospective observational studies (Table 1). The underlying inclusion criteria for diagnosis was malignant pleural disease in 27 studies, benign disease in one, and one study included patients with both benign and malignant disease. All studies included large-volume drainage of pleural fluid as part of the protocol (via either therapeutic thoracocentesis, indwelling pleural catheter, chest drain, thoracoscopy, or a combination). Twelve studies were on pleurodesis, seven compared IPC with pleurodesis, seven were on IPC management strategies, two were on intrapleural therapy, and one was on therapeutic thoracocentesis.

**Table 1:** A summary of papers included in the review. RCT: randomised controlled trial; PCS: prospective controlled study; IPC: indwelling pleural catheter; MPE: malignant pleural effusion; ICD: intrathoracic chest drain; VASD: visual analogue scale for dyspnoea; mBorg: modified Borg scale; EORTC QLQ-C30: European Organisation for Research and Treatment of Cancer Quality of Life Questionnaire-C30; VAS: visual analogue scale; SF-36 RAND Corporation: 36-Item short-form survey; QLQ-LC13: Quality of Life Questionnaire lung cancer module; QoL: quality of life; FACIT Pal: functional assessment of chronic illness therapy-palliative care scale; ADL: activities of daily living; WHO-QoL BREF: World Health Organization quality of life: brief version; mMRC: modified Medical Research Council dyspnoea scale; FACT-G: functional assessment of cancer therapy-general; CRQ: chronic respiratory disease questionnaire; SF-6D: short-form 6 dimension

Authors	Study type	Intervention	PROMs used	Duration of data points
Psallidas et al. [9]	RCT	Ultrasound-guided vs. standard care for ICD and pleurodesis for MPE	VASD, EQ-5D-5L	0, 1, and 3 months
Shrager et al. [10]	RCT	Silver nitrate IPC vs. normal IPC for MPE	VAS pain, mBorg, and EQ-5D-5L	0, 1 months
Walker et al. [11]	RCT	IPC vs. therapeutic aspiration for benign effusion	VASD, EQ-5D-5L	VASD: 0, daily over 12 weeks, EQ-5D-5L 0, 4, 8, and 12 weeks
Zhang et al. [12]	RCT	Topical mirabilite/rhubarb vs. control with intrathoracic chemotherapy and ICD for MPE	EORTC QLQ-C30	0, 14, and 28 days
Bhatnagar et al. [13]	RCT	Thoracoscopic poudrage vs. ICD and talc pleurodesis for MPE	VASD, VAS pain, EQ-5D-5L, and SF-36	0, 30, 90, and 180 days
Yo et al. [14]	RCT	Thoracoscopic pleurodesis with talc vs. doxycycline vs. silver nitrate for MPE	mBorg	unclear
Bhatnagar et al. [15]	RCT	Talc pleurodesis vs. placebo via IPC for MPE	VASD, VAS pain, QLQ-C30, and EQ-5D-5L	0, 14, 28, 42, and 56 days
Muruganandan et al. [16]	RCT	Daily vs. alternate day IPC drainage for MPE	VASD, VAS pain, and EQ-5D-5L	0, daily VAS diary, 0.5, 1, 2, 3, 4, 5, and 6 months
Petrella et al. [17]	Controlled trial	PleurX vs. Pleurocath with talc poudrage for MPE	VASD, VAS pain, and QLQ-C30, QLQ-LC13	Fay 1
Saka et al. [18]	PCS	ICD and talc pleurodesis for MPE	VAS pain	0, 30 days
Boshuizen et al. [19]	RCT	IPC vs. ICD and talc pleurodesis for MPE	mBorg	0, 6 weeks
Thomas et al. [20]	RCT	IPC vs. ICD and talc pleurodesis for MPE	VASD, EQ-5D-5L, and VAS QoL	0, 1, 30 days, 6 months, and 1 year
Wahidi et al. [21]	RCT	Daily vs. alternate day IPC drainage for MPE	SF-36	0, 2, and 12 weeks
Walker et al. [22]	PCS	Patient-centred outcomes for IPC vs. thoracoscopy vs. ICD and talc pleurodesis in MPE	FACIT-Pal, London Chest ADL Scale	0, 2, and 6 weeks
Andrade Neto et al. [23]	RCT	1% vs. 2 % iodopovidone pleurodesis via ICD for MPE	WHO-QoL BREF	0, 4 weeks
Lorenzo et al. [24]	PCS	IPC for MPE	QLQ-C30, QLQ-LC13	0, 30, and 60 days
Ost et al. [25]	RCT	VATS talc pleurodesis	mBorg	Pre and postoperative
Basso et al. [26]	PCS	IPC for MPE	mBorg, SF-6D	0, 2, and 4 weeks, followed by monthly to 1 year
Davies et al. [27]	RCT	IPC vs. ICD and talc pleurodesis for MPE	VASD, VAS pain, QLQ-C30	0, 1.5, 3, and 6 months
Demmy et al. [28]	RCT	IPC vs. ICD and talc pleurodesis for MPE	Condensed Memorial Symptom Assessment scale, a non-standard measure of breathlessness	0, 7, and 30 days
Fysh et al. [29]	PCS	IPC vs. ICD and talc pleurodesis for MPE	VASD	0, daily diary for 7 days
Cartaxo et al. [30]	PCS	Large-volume thoracocentesis for pleural effusions	mBorg	day 2
Mohsen et al. [31]	RCT	Thoracoscopic poudrage vs. iodine pleurodesis via ICD for MPE	mMRC	Not clear
Reddy et al. [32]	PCS	Thoracoscopy talc poudrage for MPE	mBorg	Not clear
Terra et al. [33]	PCS	ICD and silver nitrate pleurodesis for MPE	VAS pain, WHO-QoL BREF	1 month
Terra et al. [34]	RCT	Thoracoscopic talc poudrage vs. talc pleurodesis for MPE	SF-36, WHO-QoL BREF	Not clear
Dresler et al. [35]	RCT	Thoracoscopic talc poudrage vs. ICD and talc pleurodesis for MPE	VAS pain, QLQ-C30	Not clear
North et al. [36]	RCT	Large-volume thoracocentesis and intrapleural methylprednisolone for MPE	VASD, FACT-G	2 weeks
Putnam et al. [37]	RCT	IPC vs. ICD and doxycycline pleurodesis for MPE	mBorg, CRQ	0, 4, 8, and 12 weeks

A total of 16 PROMs were used in these papers (Table 2). PROMs were the primary outcome in eight studies and a secondary outcome in the remaining 21 studies (Table 1). Between one and four PROMs were used in each study, with a median of two. There was significant variability in time points at which PROMs were recorded, but these were generally recorded before drainage and at one to four time points after drainage (Table 2). The shortest duration of follow-up was a single measurement at 48 hours and the longest was monthly measurements for one year. It was not clear when PROMs were recorded in six studies. Some PROMs were used at inappropriate time points, e.g., one study used the EORTC QLQ-C30 one day after the intervention to assess the benefit of intervention but this PROM assesses the patient over the past two weeks [17]. Only 13 studies gave rates of missing data. Generally, in these studies, initial data completeness was good but decreased over time due to death or withdrawal. No studies commented on missing data due to patients not wanting to complete PROMs. Generally, available data on the results of PROMs was poor, with some papers not reporting on changes from baseline to follow-up and others stating there was no change in outcome without providing specific figures.

**Table 2:** A summary of PROMs used in the reviewed studies. PROM: patient-reported outcome measure; VASD: visual analogue scale for dyspnoea; mBorg: modified Borg scale; EORTC QLQ-C30: European Organisation for Research and Treatment of Cancer Quality of Life Questionnaire-C30; VAS: visual analogue scale; SF-36 RAND Corporation: 36-Item Short Form Survey; QLQ-LC13: Quality of Life Questionnaire Lung Cancer Module; QoL: quality of life; FACIT Pal: functional assessment of chronic illness therapy-palliative care scale; ADL: activities of daily living; WHO-QoL BREF: World Health Organization quality of life: brief version; mMRC: modified Medical Research Council dyspnoea scale; FACT-G: functional assessment of cancer therapy-general; CRQ: chronic respiratory disease questionnaire; SF-6D: short-form 6 dimension

Tool (brief description)	Number of studies	Mean change from baseline to the first follow-up (95% CI)	Period	Minimal clinically important difference
VASD (non-standardised)	10	Statistically but not clinically significant improvement (7.4 mm, 95% CI = 3.2–11.6, p < 0.001 [9]) (10.4 mm, 95% CI = 1.2–19.6, p = 0.03 [11])	Varies, usually lasts 24 hours	19 mm, defined in this patient group [4]
		Statistical and clinically significant improvement (25.5 mm, 95% CI 18.6 – 32.3, p < 0.001 [13])		
		Improvements are reported but insufficient data to calculate overall mean change [15-17,20,27,29,36]		
mBorg (visual analogue scale with anchoring words, can be used at rest, recollection of previous exercise, or during standard exercise)	8	No data on change from baseline reported [10,14,26]	Varies, can be during a standardised exercise test	Has been defined in other patient groups
		Improvement is reported but insufficient data is presented to calculate mean change [19]		
		Statistically significant improvement at rest and after a 6-minute walk (rest = 1.2, 95% CI = 2.0–0.4, p = 0.003; 6-minute walk = 2.7, 95% CI = 1.6–3.6, p < 0.001 [30])		
		Mean decrease of 3.3, insufficient data to calculate CI/p-value [32]		
		Mean decrease at rest 1.1 (95% CI = 0.66–1.5, p < 0.001), after exercise 2.5 (95% CI = 2.0–3.0, p < 0.001) [37]		
		Significant improvement (2.31, 95% CI = 2.66–1.95, p < 0.001) [25]		
mMRC (modified Medical Research Council score), Likert scale	2	Significant improvement in the mMRC score of the mean (SD) 4.2 (0.8) to 2.7(1.0), p < 0.001 [26]	Not specified	Has been defined in other patient groups
		Decrease in the mMRC score but insufficient data to calculate mean change [31]		
Non-standard measure of breathlessness, Likert scale	2	Improvement in breathlessness in both studies, unable to calculate the change in mean (Likert scale) [22,28]	Not specified	Not defined
VAS pain	9	No difference but no figures are given [10]	Varies	16 mm, defined in this patient group [38]
		No significant difference (mean increase = 1.6 mm, 95% CI = -2.7–6.0, p = 0.47) [13]		
		Insufficient data to calculate [15-17,39]		
		Mean decrease of 6.5 mm but insufficient data to calculate 95% CI/p-value [18]		
		Non-clinically significant decrease in chest pain in both groups (8.2 mm, 95% CI = 0.3–16.2 mm in the IPC group, 4.4 mm, 95% CI = 3.8–12.6 mm, no p-values given) [40]		
		No significant difference is reported, and insufficient results are given to calculate mean change [33]		
VAS QoL	1	Improvement is reported in one study, but no values are given [29]	Today	Not defined
QLQ-C30 [41] (30 questions, validated for use in cancer, five functional scales (physical, role, cognitive, emotional, and social), three symptom scales (fatigue, pain, and nausea/vomiting), 6 single items, global health status/QoL scale (Likert scales)	6	Improvement in physical, social, fatigue and global health but baseline values not given [12]	Past week	Defined generally for patients with cancer as about 5–10 [42]
		No comparison of baseline and follow-up reported [15]		
		Not measured at baseline [17]		
		Improvement in mean global health status 9.21 (95% CI = -1.55–19.98, p = 0.09), functional scale 6.93 (95% CI = -0.77–14.63, p = 0.076), and symptoms -9.59 (95% CI = -18.13 to -1.03, p = 0.03) [24]		
		Improvement in global health status in both groups (18.3 in the IPC group, 7.1 in the talc group, insufficient data to calculate 95% CI and p-value) [27]		
		No difference is reported but no values are reported [35]		
EQ-5D-5L [43]: The utility component consists of five items with a Likert scale and VAS health	7	No difference in utility (mean difference = 0.015, 95% CI = -0.036–0.066), improvement in VAS health (mean difference = 7.11, 95% CI = 3.3–10.9, p = 0.0003) [9]	Today	Has been defined in other patient groups
		Insufficient data to calculate [10,11,13,15,16,20]		
RAND 36-Item Short Form survey (SF-36) [44]: assesses eight health concepts: physical functioning, bodily pain, role limitations due to physical health problems, role limitations due to personal or emotional problems, emotional well-being, social functioning, energy/fatigue, and general health perceptions	3	Mean change in utility score 0.03 (95% CI = 0.02–0.04, insufficient data to calculate p-value) [13]	Mostly ‘Now’, 1 item compared to last year	Has been defined in other patient groups
		Mean change in VAS health 0.8 (95% CI = 0.2–1.4, insufficient data to calculate p-value) [21]		
		The article states no difference in score but no data is given [34]		
WHO-QoL BREF [45] (looks at domains of physical health, psychological, social relationships, and environment). Positive change is an improvement	3	Mean change for physical score 11.3 (95% CI not given, p < 0.01); environmental score 7.8 (95% CI not given, p = 0.03) [33]	Last 2 weeks	Has been defined in other patient groups
		Articles state no difference in score but no data is given [23,34]		
EORTC QLQ-LC13 [46] (additional module to QLQ-C30 for lung cancer with 13 items including three questions on breathlessness)	2	Mean change for symptom scales -6.5 (95% CI = -14.7–1.7, p = 0.11), mean change in breathlessness items -21.6 (95% CI = -33.8 to -9.4, p < 0.01) [24]	Past week	Defined for patients with lung cancer
		No baseline measurement [17]		
FACIT-Pal [47] (46 point questionnaire aimed at palliative care, five subdomains)	1	Mean change per day 0.25 (95% CI = 0.13–0.36, p < 0.01) [22]	Past 7 days	Not defined
London Chest ADL scale [48] (level of breathlessness while performing 15 activities)	1	Mean change per day -0.11 (95% CI = -0.18 to -0.4, p < 0.01) [22]	Last few days	Has been defined for patients with COPD
FACT-G [49] (27-item questionnaire, measures four domains in cancer patients, including physical, social, emotional, and functional well-being)	1	Mean change at 2 weeks 1.7 (insufficient data provided in the article to calculate 95% CI or p-value, the article states not significant) [36]	Past 7 days	Defined in patients with cancer
CRQ [50] (20 items, with four domains)	1	Mean change at 30 days of 5.3 (95% CI = 3.6–7.0, p < 0.01) [37]	Last 2 weeks	Defined in patients with other chronic respiratory disease
Condensed Memorial Symptom Assessment scale [51] (14 items, three subscales)	1	Data not provided in the article [28]	Last week	Not defined
SF-6D (6 items, Likert scale)	1	Mean change in utility 0.023 (95% CI = -0.004–0.05, p = 0.1) [26]	Not stated	Defined in other patient groups

The most common PROMs used in 22/29 studies were unidimensional measures of breathlessness, namely, VASD, mBorg, mMRC, and non-standard unidimensional measures of breathlessness which appeared to have been devised specifically for the published study (Table 2). The VASD and mBorg are both VAS, with the mBorg having the addition of anchoring words. The mMRC and the non-standard measures of breathlessness used are Likert scales. In 16/17 studies with sufficient data, these studies showed a significant improvement in breathlessness. In the one study which did not show an improvement, VASD was measured two weeks following thoracocentesis, a time point at which it would not be expected to determine maintained benefit.

Other unidimensional scales used were VAS for pain and quality of life. The VAS pain was used in nine studies, but only one showed a statistically significant decrease in pain following intervention (Table 2) [27]. This was a mean decrease of 8.2 mm in one group, but this was less than the 13 mm which has been used as the MCID for the VAS pain in patients with pleural disease [50]. A VAS for quality of life was also used in one study and showed an improvement.

Other PROMs used were multidimensional and included assessment of a range of symptoms, activity, and quality of life, as well as other dimensions, such as emotions. The results of studies using these PROMs are summarised in Table 2. Of these, the QLQ-C30 and EQ-5D-5L were the most used. For the QLQ-C30, the global health status improved in all three of the studies that reported this result. Two studies reported benefits in some subdomains (physical, social, fatigue) but did not report benefits in other subdomains. It is unclear whether only positive results were reported. The EQ-5D-5L showed improvements in the VAS health scale in three of seven papers but no changes in the index score in three of seven papers. Other multidimensional PROMs were used in three or fewer studies (Table 2).

VAS scores for dyspnoea and pain are the only PROMs that have specifically been validated in pleural disease to allow MCID definition. Mishra et al. found the VASD MCID was 19 mm which was the mean decrease in VASD in patients reporting a “small but just worthwhile decrease” in their dyspnoea on a seven-point Likert scale [4]. The VAS MCID has been found to be 16 mm in patients undergoing pleural procedures [38].

Discussion

Relief of symptoms, improvement in activity levels, and improvement in quality of life are important outcomes for patients and clinicians when draining a pleural effusion. Clinical trials to determine the most appropriate way to drain an effusion need to evaluate these symptoms. Our results demonstrate that a range of different PROMs are used to assess these symptoms in published studies. These PROMs assess a range of outcomes, most commonly breathlessness. Most studies used more than one PROM, demonstrating that there is no single PROM which encompasses all the important outcomes in these patients. Our results found that unidimensional measurements of breathlessness were responsive to pleural effusion drainage, regardless of which PROM was chosen. Multidimensional PROMs often asked about variables which were not relevant to this patient population and appeared to only be responsive in certain subscales.

Although these PROMs have been used in clinical trials, only the VASD has been validated for use in patients with pleural effusions and has a defined MCID for this population [2,4]. The limitation of the VASD is that it is not standardised. The use of unvalidated PROMs may mean that the PROMs used are unresponsive to pleural fluid drainage and lead to false-negative results. Likely, these PROMs are not responsive because they do not ask about variables which respond to pleural fluid drainage, or they include other factors not relevant to patients with pleural effusion (e.g., bowel symptoms) that dilute any signal. Furthermore, some of these PROMs were developed for other uses and are being applied to patients with pleural effusion without proper validation, including assessment of face validity, e.g., the EORTC QLQ-30 was designed to assess the well-being of cancer patients and some items are not relevant to patients with pleural effusion.

There were problems noted with the reporting of PROMs in these studies. Reporting of when PROMs were recorded was poor in a minority of studies. Only around half of the studies reported rates of missing data for PROMs. This may lead to bias in reporting the results. Generally, there was a lack of detail reported in the results of PROMs. However, completion rates (when given) were good, suggesting that generally PROMs are acceptable to patients with pleural disease.

Although we included the EQ-5D-5L in this systematic review as a PROM, it is a key component of cost-utility analysis and is frequently included in studies as a standardised measure of health status for economic (as well as clinical) appraisal. The index value of the EQ-5D-5L is used to calculate quality-adjusted life years and inform economic evaluations of healthcare interventions.

Some PROMs with multiple subdomains were reported as multiple separate outcomes, leading to the risk of false-positive results. Collecting data on outcomes which are not of interest puts an unnecessary burden on both patients and researchers.

Activity levels and breathlessness are inextricably linked. The experience of breathlessness depends on whether the patient is at rest or active, as well as on what activity they are doing. Conversely, patients may limit their activity to avoid breathlessness. Some PROMs (e.g., the mBorg) ask specifically about breathlessness at rest and on exertion. In contrast, the VASD asks about ‘average’ breathlessness during the day. The multidimensional PROMs generally ask about a range of activities of different levels of exertion to decrease the ceiling effect of these questions. A limitation of these questionnaires is that (except for the London ADL Questionnaire) they do not have the option to complete if the patient does not do that activity and whether they do not do that activity because they do not need to do it (e.g., they do not climb stairs because they live in a single-level accommodation) or because they are unable to do it due to breathlessness. This limits the accuracy of these PROMs.

Patients who have pleural fluid drained may increase their activity levels and, hence, their breathlessness decreases less than expected. There is a wide variation in which activities are performed by patients with effusions. Actigraphy data shows that patients with malignant pleural effusions spend about three-quarters of their time sedentary and less than 1% doing moderate-to-vigorous physical activity [51]. Therefore, items asking about strenuous activity will have little relevance to most patients with pleural effusions.

There were no studies which showed a clinically significant change in pain following intervention. This implies that chest pain does not appear to change significantly following pleural intervention (apart from the pain at the time of the procedure itself). Pain is often measured in these patients to assess the pain of the pleural procedure, pleurodesis, or to assess whether there is long-term pain following permanent drain insertion.

We found PROMS covering wide-ranging aspects of quality of life showing the inconsistency of response to pleural intervention, more often within subscales, but none of these have been specifically validated in pleural disease. Likely, some elements of these are relevant to the symptom burden of our population but either they are not being asked in the right way or any signal is lost within other questions of little consequence.

There have been previous systematic reviews and meta-analyses of response to pleural procedures in patients with pleural effusions although none focusing on PROMs. In agreement with our results, Wang et al. found that randomised controlled trials comparing talc pleurodesis with IPC showed both treatments were effective at relieving breathlessness but no difference in quality of life [52]. A network meta-analysis of interventions for the management of malignant pleural effusions also showed that these procedures are effective in relieving breathlessness [53]. A systematic review focusing specifically on the quality of life after interventions for malignant pleural effusions found, as found in this review, that there was an improvement in the quality of life following pleural intervention [54]. Therefore, our results are consistent with previous reviews of the literature.

## Conclusions

A range of PROMs are used in studies of pleural fluid drainage to assess symptoms, activity levels and quality of life. However, some PROMs used are not responsive to pleural fluid drainage and are not validated in patients with pleural effusions. Future trials should ensure that the PROMs selected are validated in this group and have a defined MCID. These results demonstrate that there is a need for standardised outcome measures in these clinical trials which are validated for patients with pleural disease, which encompass all outcomes of interest and are validated in this patient group with a defined MCID.
